# Trilobite-inspired neural nanophotonic light-field camera with extreme depth-of-field

**DOI:** 10.1038/s41467-022-29568-y

**Published:** 2022-04-19

**Authors:** Qingbin Fan, Weizhu Xu, Xuemei Hu, Wenqi Zhu, Tao Yue, Cheng Zhang, Feng Yan, Lu Chen, Henri J. Lezec, Yanqing Lu, Amit Agrawal, Ting Xu

**Affiliations:** 1 National Laboratory of Solid-State Microstructures and Collaborative Innovation Center of Advanced Microstructures, Nanjing University, Nanjing, 210093 China; 2 College of Engineering and Applied Sciences and Jiangsu Key Laboratory of Artificial Functional Materials, Nanjing University, Nanjing, 210093 China; 3 School of Electronic Sciences and Engineering, Nanjing University, Nanjing, 210093 China; 4 Physical Measurement Laboratory, National Institute of Standards and Technology, Gaithersburg, MD 20899 USA; 5 Maryland NanoCenter, University of Maryland, College Park, MD 20899 USA; 6 School of Optical and Electronic Information, Wuhan National Laboratory for Optoelectronics, Huazhong University of Science and Technology, Wuhan, 430074 China

**Keywords:** Nanophotonics and plasmonics, Metamaterials, Imaging and sensing

## Abstract

A unique bifocal compound eye visual system found in the now extinct trilobite, *Dalmanitina socialis*, may enable them to be sensitive to the light-field information and simultaneously perceive both close and distant objects in the environment. Here, inspired by the optical structure of their eyes, we demonstrate a nanophotonic light-field camera incorporating a spin-multiplexed bifocal metalens array capable of capturing high-resolution light-field images over a record depth-of-field ranging from centimeter to kilometer scale, simultaneously enabling macro and telephoto modes in a snapshot imaging. By leveraging a multi-scale convolutional neural network-based reconstruction algorithm, optical aberrations induced by the metalens are eliminated, thereby significantly relaxing the design and performance limitations on metasurface optics. The elegant integration of nanophotonic technology with computational photography achieved here is expected to aid development of future high-performance imaging systems.

## Abstract (web summary)

Inspired by the optical structure of bifocal compound eyes, the authors demonstrate a nanophotonic light-field camera with large depth of field. By using a spin-multiplexed bifocal metalens array and neural network-based reconstruction, they capture high-resolution images at centimeter to kilometer scale.

## Introduction

**Fig. 1:**
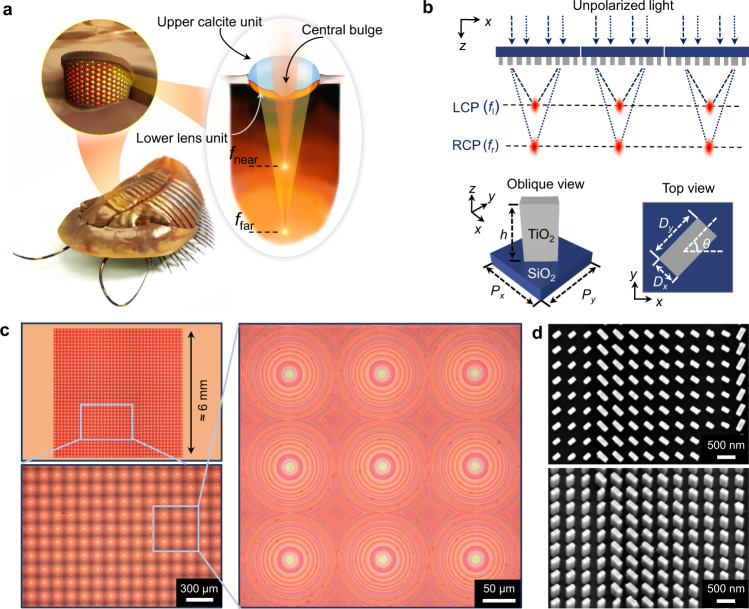
Bifocal compound eyes of trilobite *Dalmanitina socialis* and its optical analogy. **a** Conceptual sketch of extinct trilobite *Dalmanitina socialis* and its compound eyes. Each compound eye, composed of a lower lens unit and an upper lens unit with central bulge, can simultaneously focus the incident light to near and far point, similar to a coaxial bifocal lens. **b** The bioinspired photonic spin-multiplexed metalens array. The unit cell of metalens array is composed of rectangle amorphous TiO_2_ nanopillar sitting on a SiO_2_ substrate with ${P}_{x}={P}_{y}=450$ nm, and height $h=600$ nm. **c** Optical microscope image of the fabricated metalens array. The right panel shows a zoomed-in image of 3 × 3 submetalens array. **d** The scanning electron microscopy (SEM) images show the top view and oblique view of the TiO_2_ nanopillars.

Trilobites were one of the most thriving early animals that first appeared in the Cambrian^1–4^. Although they have been extinct for several hundred million years, investigations of their fossil remains reveal that they are one of the earliest arthropods with compound eyes^3,4^. Among the various trilobite groups, one trilobite, *Dalmanitina socialis*, possessed a unique visual system with compound eyes composed of two optically homogeneous lens units of different refractive indices^3,4^—an upper lens unit with a central bulge made of calcite and a lower lens unit made of an organic compound (Fig. 1a). As a result, each compound eye of *Dalmanitina socialis* is able to simultaneously focus incident light to a near and a far point, analogous to a coaxial bifocal lens, which may enable them to be sensitive to light-field information with large depth-of-field (DoF), and clearly see both close (e.g., floating preys) and distant (e.g., approaching enemies) objects in the environment. To the best of our knowledge, this type of compound-eye visual system is unique to *Dalmanitina socialis*, and is in contrast to the single focal vision system present in all-known living arthropods that exist today.

Light-field cameras could measure a rich 4D representation of light that encodes color, depth, specularity, transparency, refraction, and occlusion^5^. DoF and spatial resolution are two key system parameters in light-field photography^6–9^. DoF refers to the range of depth in object space over which one can obtain a clear reconstructed scene from various subimages captured on the sensor plane, whereas spatial resolution corresponds to the minimum resolvable spatial interval in the final rendered single-view image. Early designs of light-field camera utilized a microlens array placed at the focal plane of the primary lens to project rays arriving from different directions toward a corresponding point on the sensor to form a subimage^6^. Each subimage in this scheme is treated as an equivalent spatial point within the full imaging space, and while the camera can have a relatively large DoF, its spatial resolution is quite low. Another design of light-field camera is to place the microlens array slightly away from the focusing plane of the primary lens and achieve a higher lateral resolution, however, this comes at the expense of fewer number of resolvable directions and a reduced DoF^7^. Recently, a multifocal microlens array, in which individual lens units with different focal lengths are spatially interlaced next to each other, is proposed to extend DoF in light-field imaging^8^—however, this is also achieved at the expense of spatial resolution. Therefore, achieving large DoF without compromising spatial resolution is a challenge in light-field photography.

Existing works on conventional imaging have been exploited to extend the depth-of-field through shrinking the aperture size, focal sweeping^10^, wavefront coding^11–13^, and stacking of transparent photodetectors^14^. However, these methods have to make a compromise between imaging performance (e.g., light throughput, time resolution, color imaging capability, and imaging fidelity) and DoF. Recently, leveraging the ability of metasurface optics to provide unprecedented functional characteristics^15–25^, many outstanding sensors and imaging systems have been proposed, such as depth sensing^26,27^, full Stokes polarization imaging^28–30^, quantitative-phase imaging^31^ and angle-sensitive photodetector for lensless imaging^32^. In the field of light-field imaging, achromatic metalens used for full-color light-field imaging has also been demonstrated^33^. These works highlight the potential of metasurface optics in creating advanced imaging systems.

Here, inspired by the optical structure of bifocal compound eyes found in *Dalmanitina socialis*, we demonstrate a nanophotonic camera incorporating a spin-multiplexed metalens array able to achieve high-resolution light-field imaging with a record DoF. The proposed spin-multiplexed metalens array provides two completely decoupled transmission modulation to a pair of orthogonal circular polarization input, and thus can simultaneously capture light-field information for both close and distant depth ranges while maintaining high lateral spatial resolution. Consequently, light-field information over large DoF can be computationally reconstructed from a single exposure. In addition, inspired by the biological neural aberration compensation mechanism, we introduce a distortion-correction neural network to eliminate the aberrations, which significantly relaxes the design and performance limitations on metasurface optics. As a result, the proposed camera system is capable of achieving full-color light-field imaging with a continuous DoF ranging from 3 cm to 1.7 km with close to diffraction-limited resolution. We envision that this integration of nanophotonics with computational photography may stimulate development of optical systems for imaging science that go well beyond traditional light-field imaging technology.

## Results

### Trilobite-inspired photonic spin-multiplexed metalens array

Figure 1b shows the schematic diagram of the bioinspired photonic spin-multiplexed metalens array, where each metalens is composed of an array of subwavelength TiO_2_ nanopillars. For each metalens, the incident light is assumed to be in two orthogonal spin states: |*L*〉$$=\left[\begin{array}{c}1\\ i\end{array}\right]$$ and |*R*〉$$=\left[\begin{array}{c}1\\ -i\end{array}\right]$$, where |*L*〉 and |*R*〉 denote left-circularly polarized (LCP) and right-circularly polarized (RCP) states, respectively. In order to achieve spin-multiplexed bifocality, the metasurface can be described by a Jones matrix $J(x,y)$ that simultaneously satisfies *J*(*x*,*y*)|*L*〉 = ${e}^{i{\varphi }_{l}(x,y)}$|*R*〉 and*J*(*x,y*)|*R*〉 = ${e}^{i{\varphi }_{r}(x,y)}$|*L*〉 ^20^, where ${\varphi }_{l}(x,y)$ and ${\varphi }_{r}(x,y)$ denote two spin-dependent, uncorrelated phase profiles encoded on the metasurface to focus incident light at different focal lengths. Under these conditions, the Jones matrix $J(x,y)$ can be expressed as

1 $$J\left(x,y\right)=\left[\begin{array}{cc}{e}^{i{\varphi }_{l}\left(x,y\right)} & {e}^{i{\varphi }_{r}\left(x,y\right)}\\ {-{ie}}^{i{\varphi }_{l}\left(x,y\right)} & {{ie}}^{i{\varphi }_{r}\left(x,y\right)}\end{array}\right]{\left[\begin{array}{cc}1 & 1\\ i & -i\end{array}\right]}^{-1},$$

and phase profiles ${\varphi }_{l}(x,y)$ and ${\varphi }_{r}(x,y)$ rely on the following form:

2 $${\varphi }_{l,r}\left(x,y\right)=\frac{2{{{{{\rm{\pi }}}}}}}{\lambda }\left({f}_{l,r}-\sqrt{{x}^{2}+{y}^{2}+{f}_{l,r}^{2}}\right),$$

where ${f}_{l,r}$ denotes the desired focal lengths for the two polarization states, and $\lambda$ is the wavelength of operation. The analytical solutions extracted from the eigenvalues and eigenvectors of Jones matrix in Eq. (1) determine the required spatially varying propagation phase and geometric phase to be provided by each nanopillar of the metasurface. Therefore, it is imperative to find a set of TiO_2_ nanopillars with optimized major (minor) axis length ${D}_{x}({D}_{y})$ and orientation angle $(\theta )$ to form the spin-multiplexed metasurface. The details of design are provided in Supplementary Information Section I.

For proof-of-concept demonstration, we fabricate a 39 × 39 array of TiO_2_ metalenses with a fill factor of 100% achieved by close-packing individual square-shaped metalenses in a square lattice (Fig. 1c). Each metalens has a side length *d* = 150 μm and consists of ≈110,000 rectangular TiO_2_ nanopillars. Since the green channel in the Bayer filter array of the image sensor is twice as dense as the red or the blue channels, and after taking into account the sensor’s spectral sensitivity, here we perform the initial phase design of metalens array at a green wavelength of 530 nm. Two focal lengths, ${f}_{l}$ = 900 μm and ${f}_{r}$ =  1250 μm, respectively, for incident LCP and RCP light at 530 nm, are judiciously chosen according to the targeted DoF of the imaging system. Figure 1d shows the scanning electron microscope (SEM) image of the fabricated TiO_2_ metalens. The top-view and perspective-view images show well-defined nanopillars, exhibiting great fidelity to our design. The detailed fabrication process is described in “Methods”.

**Fig. 2:**
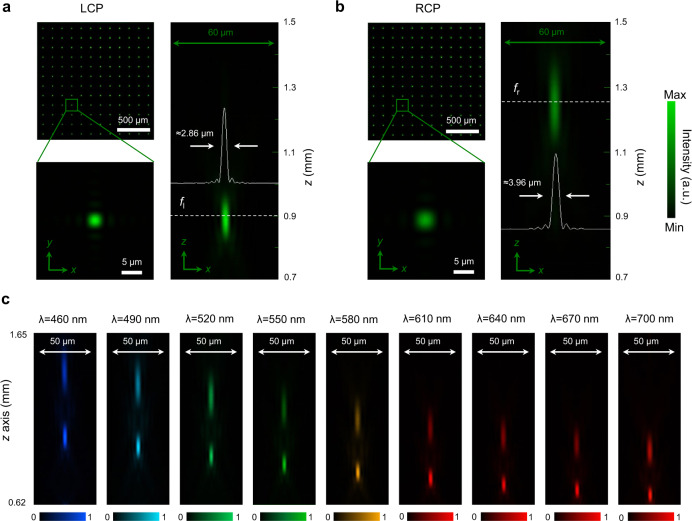
Measured intensity distributions of photonic spin-multiplexed metalens arrays. Focal spots in the $x$-$y$ and $x$-$z$ plane for (**a**) LCP and (**b**) RCP incident light at the wavelength of 530 nm. For ease-of-viewing, here we show an array of 12 × 12 focal spots (top left). The solid white lines show the horizontal cuts of the intensity distributions of focal spots. **c** Dispersion of a single submetalens illustrated by metalens focusing at different focal lengths for the wavelength spanning from 460 nm to 700 nm. The incident light is linearly polarized.

To characterize the optical performance of the metalens array, a collimated circularly polarized laser beam at a free-space wavelength of 530 nm illuminated the metasurface at normal incidence. A schematic diagram of the measurement setup is shown in Supplementary Fig. S3. As expected, the focal length is strongly dependent on the polarization of incident light. The focal length for LCP and RCP is measured as ${f}_{l}$  = (895 ± 6) μm and ${f}_{r}$ = (1243 ± 9) μm, which agree well with the design values. The uncertainties are standard deviation for repeated measurements. The light-intensity distribution collected at two focal planes ${f}_{l}$ and ${f}_{r}$ is depicted in Fig. 2(a, b). From the normalized intensity distribution of the magnified single-focused spot at *x–y* and *x–z* planes, the measured full-widths at half-maximum (FWHM) of focal spots for LCP and RCP light are respectively (2.86 ± 0.04) μm and (3.96 ± 0.06) μm, close to the theoretical diffraction-limited FWHM of 2.83 μm and 3.92 μm, respectively.

In our design, the metalens array exhibits efficient chiral bifocality over a free-space wavelength range spanning from 460 nm to 700 nm, realizing broadband photonic spin-multiplexing in the visible. The chromatic dispersion of the metalens is the same as that expected from a conventional diffractive optic, where the device exhibits wavelength-dependent focal shifts, as shown in Fig. 2c. The average transmission efficiency over the entire bandwidth is (72 ± 1.5)% for the unpolarized incident light. The average focusing efficiency is (43.6 ± 1.6)% and (42.8 ± 1.2)% for the LCP and RCP incident light, respectively. The uncertainties in efficiency measurements are standard deviation for repeated measurements. Here, the focusing efficiencies are calculated as the ratio of the power passing through a 10-μm-diameter pinhole place at the focus to the total power incident on the submetalens. The focusing efficiency of the proposed metalens can be substantially improved by (a) further optimizing the nanofabrication process; and (b) employing machine-learning techniques to optimize the design-parameter space of nanostructures (height, shape, and lateral dimensions of the nanostructures, constituent material, and lattice constant). It is worth noting that the photonic spin-multiplexed metalens proposed here can provide two completely decoupled wavefront modulations to a pair of orthogonal circular polarization input and maintain complete spatial frequency information when used in imaging, which cannot be achieved by employing spatially multiplexed multifocal lenses. The mechanism employed here provides a feasible way to break the constraint between DoF and spatial resolution.

### Construction of metalens-based light-field imaging system

**Fig. 3:**
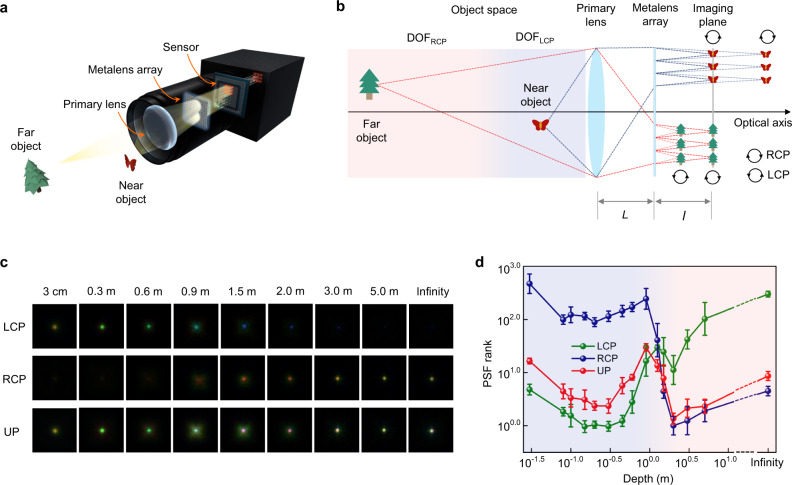
Light-field imaging system enabled by the spin-multiplexed metalens array. **a** Conceptual sketch of the proposed light-field imaging camera. **b** Schematic diagram of the working principle of the system with metalens array achieving spin-dependent bifocal light-field imaging. Either the LCP component of close object or the RCP component of distant object could be focused well on the identical imaging plane. The nominal distance between the primary lens and metalens array is $L=47.5$ mm. The nominal distance between the imaging plane and metalens array is $l=0.83$ mm. The focal length and aperture size of the primary lens is $F=50$ mm and $D=6$ mm, respectively. **c** The captured PSFs at different depths for LCP, RCP, and UP (unpolarized) incident light. **d** Demonstration of working range for different polarization states. The light-blue region and light-red region represent the working range of LCP and RCP components, respectively. The vertical axis represents the PSF ranks, for which the smaller value corresponds to better imaging quality. The uncertainties are standard deviation for repeated measurements (six in total).

Using the spin-multiplexed metalens array, we construct a proof-of-concept light-field camera exhibiting extreme DoF and high spatial resolution, breaking the trade-off between these two figure-of-merits in conventional light-field imaging systems^5–8^. Through a rigorous optical design process, the DoFs of the two bifocal channels are seamlessly connected, i.e., the far boundary of DoF from LCP and near boundary of DoF from RCP are connected with each other to form a continuous DoF. A schematic diagram depicting the metalens array, placed behind the primary lens, to capture subimages of the various scenes and image them on the sensor plane, is shown in Fig. 3a. For ease of illustration, here we plot the ray tracing of monochromatic light and ignore the chromatic dispersion of the metalens array. The focal length of the metalens can be simply switched by flipping the chirality of the incident light, producing different DoF range in the object space. For close objects in the depth range, DoF_LCP_ (e.g., “butterfly” in Fig. 3b), the LCP components from the object are well focused on the sensor plane. Conversely, for distant objects in the depth range, DoF_RCP_ (e.g., “tree” in Fig. 3b), the RCP components are focused on the sensor plane. Since light originating from most nature scenes is either unpolarized or partially polarized and can be decomposed into LCP and RCP states, the system allows both close and distant objects to be simultaneously projected, focused, and imaged on the sensor plane.

The forward imaging process of the designed optical system can be modeled using the Rayleigh–Sommerfeld diffraction formula

3 $$U\left(x,y,{z}_{i},\lambda \right)=\; \left\{\left[U\left(x,y,{z}_{o},\lambda \right)* h\left(x,y,A,\lambda \right)\right] \right.\\ \cdot \left. \Phi \left(x,y,\lambda \right)* h\left(x,y,L,\lambda \right)\right\}\cdot \varphi \left(x,y,\lambda \right)* h\left(x,y,l,\lambda \right),$$

where $U\left(x,y,{z}_{o},\lambda \right)$ and $U\left(x,y,{z}_{i},\lambda \right)$ are the complex amplitudes at the object plane ${z}_{o}$ and image plane ${z}_{i}$, respectively. $h\left(x,y,A,\lambda \right)$, $h\left(x,y,L,\lambda \right)$, and $h\left(x,y,l,\lambda \right)$ are the free-space propagation functions from the object to the primary lens, from the primary lens to the metalens, and from the metalens to the imaging plane, respectively. $A$, $L$ and $l$ denote the corresponding distances along the propagation ($z$) direction (Fig. 3b). $\Phi \left(x,y,\lambda \right)$ and $\varphi \left(x,y,\lambda \right)$ are the phase profiles of the primary lens and the metalens, respectively. The point spread function (PSF) is the response of an imaging system to a point source or point object. Using Eq. 3, the point spread function (PSF) of the proposed light-field imaging system is calculated as

4 $${{{{{\rm{PSF}}}}}}=\int {H(\lambda )\left|U\left(x,y,{z}_{i},\lambda \right)\right|}^{2}d\lambda$$

To take the chromatic effect into account, here the PSF accumulates images of point light source with different visible wavelengths. $H(\lambda )$ is the wavelength response function of the imaging system, which takes both the spectral response of the image sensor and the polarization conversion efficiencies of the selected nanostructures into consideration. Since PSF is the spatial version of the optical transfer function (OTF), here we evaluate the performance of the imaging system with a PSF rank metric ${{{{{{\rm{PSF}}}}}}}_{{{{{{\rm{rank}}}}}}}=\mathop{\sum}\limits_{\omega }\frac{{\sigma }^{2}}{{\left|{K}_{\omega }\right|}^{2}+\frac{{\sigma }^{2}}{{S}_{\omega }}}$^34^, where ${{{{{\rm{\sigma }}}}}}$,${{{{{\rm{\omega }}}}}}$, ${K}_{\omega }$, ${S}_{\omega }$ denote the noise level, spatial frequency, Fourier transform of the PSF, and the average power spectra of the captured image, respectively. A smaller ${{{\mbox{PSF}}}}_{{{{{{\rm{rank}}}}}}}$ corresponds to a higher imaging quality. Using a ${{{\mbox{PSF}}}}_{{{{{{\rm{rank}}}}}}}$ based metric, we choose an optimal set of physical parameters within the limits of three design constraints of the optical system: (i) Focusing constraint: that the system is able to focus at infinity; (ii) Repetitive rate constraint: that the repetitive rate of a scene from close to distant should be at least 3 for accurate disparity estimation; (iii) DoF touch constraint: that the DoF of LCP and RCP light chirality should be seamlessly connected. Given the limited parameter space and PSF rank requirements for the imaging system, a set of eligible parameters are chosen for the optimized bifocal light-field camera (Supplementary Information Section II).

To validate the imaging performance over an extreme DoF, we first measure the PSF of the optical system. A test target, made of a 100-μm-diameter pinhole on an opaque film serving as a point light source, is illuminated with white light as it is gradually translated from $z=3\,\,{{{{{\rm{cm}}}}}}$ to infinity. Here the infinity depth is realized by collimating the white light emitted from a point source using an optical collimator. As shown in Fig. 3c, under LCP illumination, the image of the point source is focused on the sensor at the near-depth range while the far-depth range is out of focus. Instead, when the incident light is switched from LCP to RCP, the image of the point source is focused on the sensor at the far-depth range, while the near depth range is out of focus. By combining the two polarization channels, both close and distant object information can be simultaneously recorded on the imaging sensor. Note that the colors in the measured PSF images mainly originate from the inherent chromatic dispersion associated with the metalens. To quantify the performance of the imaging system, we evaluate ${{{\mbox{PSF}}}}_{{{{{{\rm{rank}}}}}}}$ according to the repeated measurement data (Fig. 3d). The ${{{\mbox{PSF}}}}_{{{{{{\rm{rank}}}}}}}$ of the LCP component exhibits a small value in the near-depth range (from ≈3 cm to $\approx$ 2 m), whereas, ${{{\mbox{PSF}}}}_{{{{{{\rm{rank}}}}}}}$ of the RCP component becomes small for far-depth range (from $\approx$ 2 m to infinity). As a result, when used in ambient environment with natural sources of light, the proposed light-field imaging system is expected to have a relatively small ${{{\mbox{PSF}}}}_{{{{{{\rm{rank}}}}}}}$ over an extreme DoF range, which satisfies the imaging requirement.

### Reconstruction algorithm based on multiscale convolutional neural network

The singlet metalens-based imaging system proposed here suffers from various spatially nonuniform optical aberrations, mainly including chromatic and comatic aberrations introduced by the metalens^33,35–41^. In addition, the location, depth, and assembling errors of the optical system lead to serious diverse aberrations in practice. In nature, optical aberrations also commonly exist in the optics of biological visual systems, including human beings. The perceived appearance, however, does not display such aberrations due to the neural adaptation/processing-induced aberration tolerance of the visual system^42,43^. Inspired by the neural aberration compensation mechanism in biological visual system, here we employ an artificial neural network with all convolutional layers to eliminate these aberrations and generate a high-quality light-field image in a semiblind way, i.e., the method requires only a simple calibration process before training and achieves excellent robustness for the diversity and disturbance of aberrations. In practice, even after reassembling the optical system, the network works well without any recalibration or retraining. A light-weight multiscale architecture is applied to deal with the severely nonuniform and diverse aberrations of our light-field imaging system, which is complimentary to other physical aberration-correction approaches utilized in literature that rely on an expanded library composed of complex unit-cell architectures to achieve the necessary group-delay dispersion required for operation over a large bandwidth^35–39^. Our approach, instead, not only corrects for severely nonuniform and diverse optical aberrations through reliable post-processing based on artificial intelligence and machine learning, but also significantly relaxes the design requirements on achromatic metasurface optics that incorporate sometimes hard-to-fabricate unit-cell architectures, and are still plagued by small aperture size and limited focusing efficiency.

**Fig. 4:**
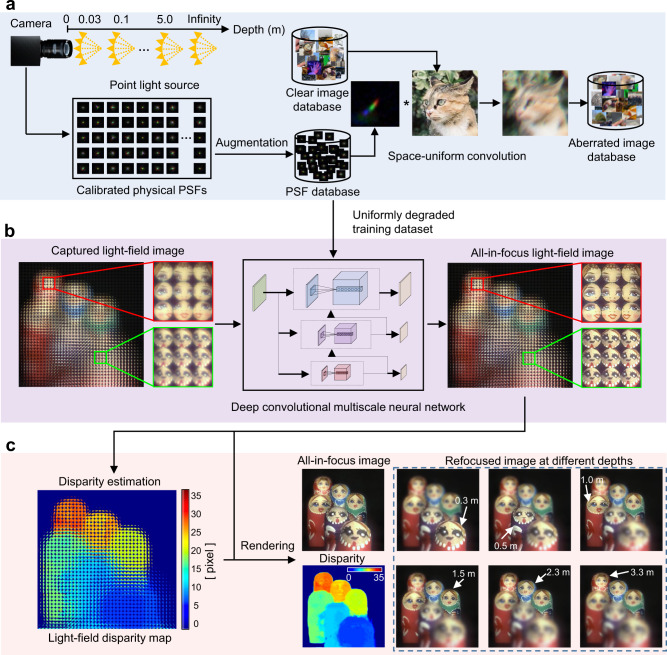
The neural network-based reconstruction algorithm processing flow. **a** PSF capture and training-data generation. **b** Aberration removal with the proposed multiscale deep convolutional neural network. The distance between the primary lens and Matryoshka nesting dolls: 0.3 m, 0.5 m, 1.0 m, 1.5 m, 2.3 m, and 3.3 m. The insets show the nearest and farthest Matryoshka nesting dolls. **c** Light-field processing based on the retrieved all-in-focus light-field images, including disparity estimation and refocusing images at different depths.

The neural network-based reconstruction algorithm processing flow is illustrated in Fig. 4. We first generate a set of training data from the physically calibrated optical aberrations of the imaging system (Fig. 4a). To obtain the real optical aberrations of the system, we capture various PSFs using a pinhole at different depths (i.e., at distance $z$ between the aperture of the primary lens and object, as shown in Fig. 4a) and lateral positions. Based on the measured PSFs, we generate a large PSF dataset with a PSF-augmentation method. Specifically, we rotated and slightly resized the PSFs calibrated at different locations and depths randomly to generate the sparse PSF basis, and generate the augmented PSF space by linearly combining the wavefront errors reconstructed from these sparse PSF basis using phase retrieval. The aberrated images for training are generated by uniformly convolving clear images with PSFs in the augmented PSF dataset. The aberrated and the corresponding clear image pairs are then used to train the aberration-correction neural network. Although the aberrations are uniform for the training data, the entire training dataset contains various forms of aberrations. This enables the neural network to autonomously handle the effects of different aberrations at any local subregion of a captured image or even other distortions in metalens system using a transfer-learning strategy^44^.

Using the generated training data, we build a multiscale convolutional neural network for aberration correction and reconstruct an all-in-focus light-field image from the experimentally captured data (Fig. 4b). The neural network is composed of multiple branches of convolutional layers with different scales of effective receptive field, which could emulate biological neural processing and aggregate information from different scales (refer to Fig. S6 for network details). To verify the feasibility of the proposed method, we experimentally design an imaging scene composed of six colorful Matryoshka dolls placed at different *z* distances. As is shown in the enlarged $3\times 3$ insets (Fig. 4b), the original light-field images of Matryoshka dolls taken by the metalens array are blurry due to aberrations. Using the trained neural network, the aberration-corrected all-in-focus image retrieved at the output of the network appears aberration-free and sharp (Fig. 4b). Besides the all-in-focus image, successive multidimensional light-field information can also be subsequently retrieved using light-field processing methods^45^, such as disparity map and refocused images at different depths (Fig. 4c). Further details of light-field processing method, including disparity estimation and rendering method, are described in Supplemental Information Section III.

Benefiting from a multiscale convolutional architecture, as well as sparse PSF-calibration and PSF-space-augmentation strategies, the proposed method does not simply work like the deconvolution-based methods^46,47^, instead, it can handle intensely nonuniform and severely diverse aberrations in a semiblind way without requiring additional PSF or location information after training. It is robust to the diversity of aberrations caused by the locations, depths, and assembly errors of the system through training with the augmented PSF space.

### Light-field imaging with extreme DoF

**Fig. 5:**
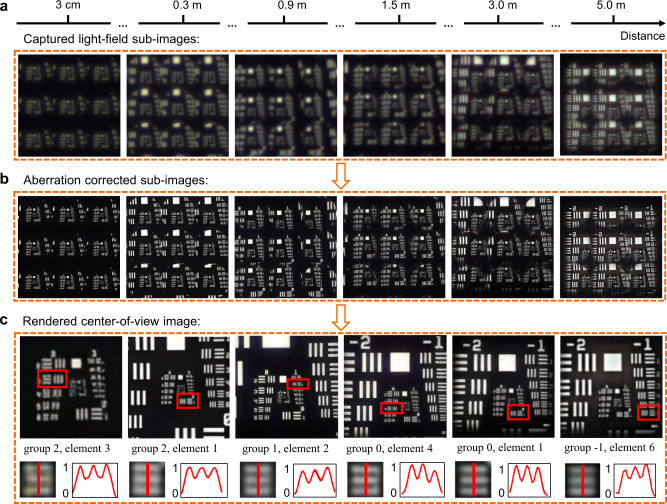
Resolution evaluation of the proposed light-field imaging system. **a** Captured subimages of a USAF 1951 resolution chart at different depths. For easy recognition, here we show the 3×3 subimages. **b** Aberration-corrected subimages. **c** Top: Rendered center-of-view images of the USAF 1951 resolution chart. Bottom: Zoom-in images and intensity cross sections of each smallest-resolvable line pair in the resolution chart.

To quantitatively evaluate the performance of the light-field imaging system and related neural network algorithm, a USAF 1951 resolution chart is placed at several different distances (from 3 cm to 5 m) away from the primary lens, and illuminated with white light. Figure 5a shows the light-field subimages of resolution chart captured by the sensor without postprocessing, whose quality is quite low. In contrast, by applying the deep-learning-based neural network correction and reconstruction algorithm, high-resolution and aberration-free subimages (Fig. 5b) and reconstructed center-of-view image (Fig. 5c) can be achieved throughout the entire working distance. From the zoom-in images and cross sections shown in Fig. 5c, the smallest line pair that could be resolved are 5.04 lp/mm (group 2, element 3) and 0.89 lp/mm (group -1, element 6) at 3 cm and 5 m, respectively. According to these rendered images of resolution chart, the corresponding angular resolution of the metalens array-based camera as a function of depth is calculated and given in Supplementary Fig. S13. Benefiting from the elegant reconstruction, the resolution of the imaging system matches well with the theoretical diffraction-limited ones calculated across the visible wavelength.

**Fig. 6:**
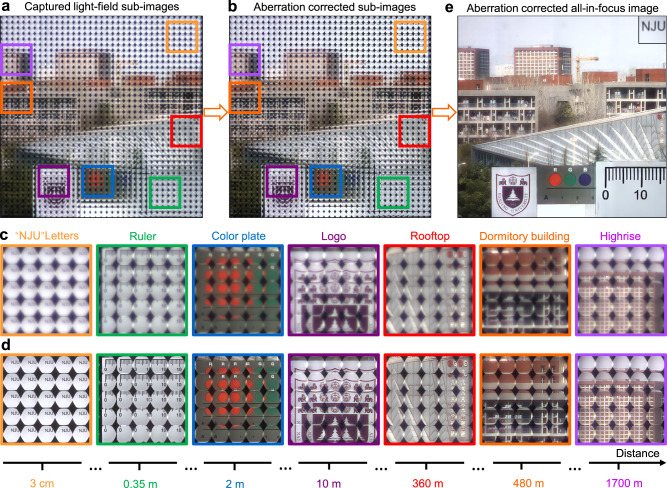
Experiment results of the light-field imaging with extreme DoF. **a**, **b** Captured light-field subimages of the whole scene under natural light (**a**) before and (**b**) after aberration correction. **c**, **d** Zoomed-in subimages of different objects corresponding to the marked ones shown in (**a**, **b**), respectively. **e** Aberration-corrected all-in-focus image after rendering. The reconstructed NJU characters have been reasonably shifted and scaled for easy viewing.

To better exhibit the capability of light-field imaging over extreme DoF by the proposed imaging system and reconstruction algorithm, we select a scene covering an enormous depth from 3 cm to 1.7 km. A piece of glass patterned with opaque characters “NJU” is placed at a depth of 3 cm away from the aperture of the primary lens, which is used as the nearest object. A ruler, a color plate, and a university logo are placed at the depth of 0.35 m, 2 m, and 10 m, respectively. The distance of white Chinese characters on the rooftop and dormitory building are approximately 360 m and 480 m, respectively. The distance of the farthest highrise is approximately 1.7 km. Figures 6a, b show the captured light-field subimages under natural light before and after neural network aberration correction, respectively. As expected, the proposed light-field imaging system enables in-focus imaging of both near and far objects. From the zoom-in subimages, it is clearly seen that the blurry effects (Fig. 6c) originating from the optical aberrations induced by the metalens array can be eliminated with the proposed aberration-correction neural network (Fig. 6d). As a result, by further using the reconstruction algorithm, a clear and sharp image of the whole scene can be obtained, covering a record depth range from 3 cm to 1.7 km (Fig. 6e). Therefore, this bioinspired nanophotonic light-field camera together with the computational post-processing not only can achieve full-color imaging with extreme DoF, but also be able to eliminate the optical aberrations induced by the meta-optics. More experimental results about light-field imaging under the LCP and RCP light are given and analyzed in Supplementary Information Section IV.

## Discussion

Inspired by compound eyes of the trilobite *Dalmanitina socialis*, we design and construct a chiral light-field camera incorporating an array of photonic spin-multiplexed bifocal metalenses. Combined with a deep-learning-based neural network reconstruction algorithm, the system provides distinct aberration-free photographic capabilities, including the ability to achieve a polarization-controllable extreme DoF imaging while maintaining high spatial lateral resolution. We envision the integrated metalens array with multifunctional response to extend the range of applications in light-field imaging systems such as consumer photography, optical microscopy, and machine vision.

## Methods

### Nanofabrication of metalens array

A fused silica substrate is spin-coated with a layer of 600-nm-thick, positive electron-beam resist. The metalens array is defined in resist using an electron-beam lithography system. This process is performed at a beam current of 2 nA and accelerating voltage of 100 kV. Then, the patterned resist is coated with a layer of TiO_2_ at a temperature of 90 °C using atomic-layer deposition (ALD). The overcoated TiO_2_ film is etched by employing an inductively coupled-plasma reactive ion etching (ICP-RIE) in a mixture of Cl_2_ and BCl_3_ gas. Finally, the sample is soaked in n-methyl-2-pyrrolidone to remove the resist.

### Characterization of metalens array

The experimental setup for measuring the focal spot of metalens array is given in supplementary Fig. S3a. A collimated laser beam passes through a polarizer and quarter-wave plate to generate circularly polarized light. The wavelength of laser is selected by using acousto-optic tunable filter system (AOTF). The microscope employs a 100 $\times$ objective with a numerical aperture (NA) of 0.8 to capture the intensity distribution of focal spots. The measured focal lengths for LCP and RCP light are shown in supplementary Figs. S3(b, c).

## Supplementary information

Supplementary Information

## Data Availability

The data that support the findings of this study are available from the corresponding author upon request.

## Code availability

The code used for analyses and figures is available from the corresponding author upon request.
