# Supplementary material for: Trilobite-inspired neural nanophotonic light-field camera with extreme depth-of-field
Source: Nat Commun. 2022 Apr 19;13:2130. doi: 10.1038/s41467-022-29568-y (PMC9019092; doi:10.1038/s41467-022-29568-y)
Supplement: Supplementary file 1 — Supplementary Information [file 41467_2022_29568_MOESM1_ESM.pdf]

Supporting information

Trilobite-inspired neural nanophotonic light-field camera with extreme depth-of-field

Qingbin Fan[1,2,3], Weizhu Xu[1,3], Xuemei Hu[1,3], Wenqi Zhu[4,5], Tao Yue[1,3], Cheng Zhang[6], Feng Yan[1,3], Lu Chen[4,5], Henri J. Lezec[4], Yanqing Lu[1,2], Amit Agrawal[4,5] and Ting Xu[1,2]

1. National Laboratory of Solid-State Microstructures and Collaborative Innovation Center of Advanced Microstructures, Nanjing University, Nanjing 210093, China
2. College of Engineering and Applied Sciences and Jiangsu Key Laboratory of Artificial Functional Materials, Nanjing University, Nanjing 210093, China
3. School of Electronic Sciences and Engineering, Nanjing University, Nanjing 210093, China
4. Physical Measurement Laboratory, National Institute of Standards and Technology, Gaithersburg, Maryland 20899, United States
5. Maryland NanoCenter, University of Maryland, College Park, Maryland 20899, United States
6. School of Optical and Electronic Information, Wuhan National Laboratory for Optoelectronics, Huazhong University of Science and Technology, Wuhan 430074, China.

I. Design of photonic spin-multiplexed metalens array

Simulation of individual birefringent nanopillars

According to Eq. (1) in the main text, the photonic spin-multiplexed metalens can be described by Jones matrix $J(x,y)$ as:

$$J(x,y)=\frac{1}{2}\begin{bmatrix} e^{i\varphi_l(x,y)}+e^{i\varphi_r(x,y)} & ie^{i\varphi_r(x,y)}-ie^{i\varphi_l(x,y)} \\ ie^{i\varphi_r(x,y)}-ie^{i\varphi_l(x,y)} & -e^{i\varphi_l(x,y)}-e^{i\varphi_r(x,y)} \end{bmatrix} \qquad (S1)$$

where $\varphi_l(x,y)$ and $\varphi_r(x,y)$ denote two spin-dependent, uncorrelated phase profiles for the metasurface to converge incident light to different focal lengths. By solving the eigenvectors and eigenvalues of the Jones matrix $J(x,y)$ for orthogonal circular polarization states, one can obtain spatial birefringent phase shifts (δ_x, δ_y) of the nanopillars as:

$$\delta_x(x,y)=\frac{\varphi_l(x,y)+\varphi_r(x,y)}{2}, \qquad (S2)$$

and

$$\delta_y(x,y)=\frac{\varphi_l(x,y)+\varphi_r(x,y)}{2}-\pi, \qquad (S3)$$

and the rotation angle θ of the nanopillars as:

$$\theta(x,y)=\frac{[\varphi_l(x,y)-\varphi_r(x,y)]}{4}. \qquad (S4)$$

Therefore, it is imperative to find a set of birefringent nanostructures with appropriate major/minor axis length (D_x and D_y) and orientation angle (θ) that satisfy the required phase to be encoded by the spin-multiplexing metasurface. The first task is to perform a parameter sweep and establish a library that determines the relation between birefringent phase shifts (δ_x, δ_y) and lateral dimensions of a nanostructure. The simulations are implemented by using a finite-difference-time-domain (FDTD) method with linearly polarized light illumination from the SiO_2 substrate side for a periodic array of anisotropic rectangular nanopillars made of TiO_2. Figs. S1(a-d) show the calculated intensity and phase shifts of the transmission coefficients by varying the lateral dimensions of a TiO_2 nanopillar ranging from 80 nm to 350 nm. According to the Eqs. (S2-S4), a subset of nanopillars is chosen as the basic building blocks for the metalens.

The transmitted wave consists of cross-polarization and co-polarization light. The focusing efficiency of the designed photonic spin-multiplexed metalens depends on the cross-polarization conversion efficiencies of selected nanopillars. Therefore, the nanostructures are chosen to maintain high transmission amplitudes for the two orthogonal linear polarizations, and simultaneously satisfy Eqs. (S2-S4). Figs. S2(a-d) show the transmission coefficients for *x*- and *y*-polarized light, and their phase difference over wide range of visible wavelengths spanning from 460 nm to 700 nm. These selected nanostructures lay a foundation for the design of high efficiency and broadband photonic spin-multiplexed metalens.

II Optical imaging system design

1. Physical parameter space

Table 1 | Parameter space for the design of imaging system

Symbol	Physical Definition
$\boldsymbol{F}$	focal length of the primary lens
$\boldsymbol{f_l}$	focal length of the metalens array corresponding to LCP light component
$\boldsymbol{f_r}$	focal length of the metalens array corresponding to RCP light component
$\boldsymbol{D}$	aperture size of the primary lens
$\boldsymbol{d}$	size of the metalens
$\boldsymbol{L}$	distance between primary lens and metalens array
$\boldsymbol{l}$	distance between metalens array and imaging sensor

2. Principle of parameter selection

In this section, we formulate the three constraints mentioned in the main text: *i.e.*, (*i*) Far/infinity focusing constraint: that the system is able to focus at infinity; (*ii*) Repetitive rate constraint: that the repetitive rate of the scene from close to distant object should be at least three for disparity estimation. Here, the repetitive rate is defined by the repetitive number of captured sub-images of the same object point; (*iii*) DoF touch constraint: that the transition of DoF for LCP and RCP light chirality should be continuous. Then we choose the physical parameters that could meet all these constraints.

As shown in Fig. S4, the imaging process could be divided into two stages, *i.e.*, the object point P_0 is first focused by the primary lens to P_1, and then P_1 is focused to P_2 by the metalens. Based on the simple lens assumption, for the first stage,

$$\frac{1}{A}+\frac{1}{L+a}=\frac{1}{F}, \tag{S5}$$

where A is the distance between P_0 and the primary lens, $L+a$ is the distance between P_1 and the primary lens, and F is the focal length of the primary lens. For the second stage,

$$\frac{1}{-a}+\frac{1}{l}=\frac{1}{f_r}, \tag{S6}$$

where l is the distance between P_2 and the metalens, a is the distance between P_1 and the metalens, and f_r is the focal length of the RCP channel. The negative sign before a is introduced since the object and image points are on the same side of the metalens.

We choose an off-the-shelf camera lens with $F = 50$ mm and D = 6 mm as the primary lens. With F chosen, the relationship between $L+a$ and A is:

$$L+a=\frac{1}{\frac{1}{F}-\frac{1}{A}}. \tag{S7}$$

The influence of A on the chosen of $L + a$ is shown in Fig. S5a. From the figure, one could determine that when the focusing distance extends from 100 m to infinity, the value of $L + a$ barely changes, staying at ≈ 50 mm. To meet the far/infinity focusing constraint, we set

$$L + a = 50\text{ mm}. \quad (S8)$$

To satisfy the repetitive rate constraint, the relationship between a and l is:

$$\frac{a}{l} = 3. \quad (S9)$$

Based on the research findings from the fossil remains of *Dalmanitina socialis*, the size of the micro-eyes of trilobite ranges from ≈ 100 μm to ≈ 200 μm (*1*), here we choose the size of the metalens d to be 150 μm to emulate the eye of trilobite.

For light-field imaging, in order to avoid overlaps among sub-images, the *f*-number of the main lens should be no less than the f-number of the metalens, i.e. $\frac{F}{D} \geq \frac{\max(f_r, f_l)}{d}$. Since the metalens has two focal lengths that correspond to different polarization channels and f_r of the RCP channel is chosen as the longer focal length, the f-number of the main lens should be no less than the metalens of the RCP channel. Here we use the following equation to match the f-number of the main lens and metalens of the RCP channel:

$$\frac{F}{D} = \frac{f_r}{d}, \quad (S10)$$

With Eq. (S10), f_r is calculated to be 1.25 mm. Then the parameters of the imaging system except f_l can be derived by solving Eqs. (S5 to S9) jointly, *i.e.*, $l = \frac{2}{3}f_r \approx 0.83\text{ mm}, a = 2f_r = 2.5\text{ mm},\ L = 50\text{ mm} - a = 47.5\text{ mm}$.

Thus far, the first two constraints are satisfied. To enable the system to focus at near objects under the DoF touch constraint, here we choose the remaining parameters f_l of the metalens for the green color channel by analyzing the imaging quality with a series of possible focal length values, ranging from 0 mm to 2.5 mm. As shown in Fig. S5b, the $\mathrm{PSF_{rank}}$ of the RCP channel (f_r =1.25 mm) exhibits a small value in the far depth range, whereas, $\mathrm{PSF_{rank}}$ of the LCP channel (f_r =0.90 mm) becomes small for near depth range. By the combination of these two DoFs from opposite light chirality, the DoF of our imaging system could theoretically be extended from 3 cm (*i.e.* $\approx 10^{-1.5}$ m) to infinity. Note that although we choose the parameters with respect to the green channel, the introduced artifacts of the other wavelengths will be resolved with the proposed aberration correction method. The physical parameters chosen for the light-field imaging system are listed in Table 2.

Table 2 | The chosen parameters of the proposed light-field imaging system.

Physical Parameters	Chosen Value (mm)
$\boldsymbol{F}$	50
$\boldsymbol{f_l}$	0.90
$\boldsymbol{f_r}$	1.25
$\boldsymbol{D}$	6.00
$\boldsymbol{d}$	0.15
$\boldsymbol{L}$	47.5
$\boldsymbol{l}$	0.83

III Neural network-based image recovery method

1. Complete process-flow of light-field image processing

Due to optical aberrations and coupling between the two polarization channels present in the proposed bifocal imaging system and the characteristics of light-field imaging, the captured light-field image suffers from much more severe spatially varying and diverse aberrations than traditional intensity imaging systems. Thus, instead of the widely used globally/locally deconvolution-based architectures, we propose a light weight multiscale convolutional neural network, which works in a semi-blind and non-deconvolutional mode, to retrieve the clear images directly. A convolutional neural network trained on the images uniformly degraded by the various of PSFs enables its handling capacities for the intense non-uniform aberrations. To simplify the calibration and facilitate the robustness of the method for diverse aberrations, we span the PSF space from few calibrated PSFs of the imaging system by augmenting the PSFs in wavefront domain. Benefiting from the multiscale convolutional architecture and a sparse PSF calibration and augmentation strategy, the proposed method is able to adaptively remove any severe non-uniform or diverse distortion at different regions automatically without requiring any additional inputs. After that, successive post-processing methods are implemented for disparity estimation and refocused image rendering.

2. Distortion removal neural network

Network architecture: The proposed neural network adopts the multi-scale structure, which is widely applied in various low-level computational tasks such as denoising, deblurring and super-resolution (*2-4*). The input signal is processed in a coarse-to-fine way with the multi-scale structure and the details of the network are shown in Fig. S6. Specifically, there are 10 res-blocks (*5*) in each scale level. Each res-block contains 64 filters with convolutional kernel of size 3×3. Zero-padding is applied to keep the size of features. In addition, global connections are introduced between the input and output of each scale, which impels the network to learn the residual information of each scale.

Loss function: We adopt the total loss combining the mean square error (MSE) and perceptual loss (*6*), *i.e.*,

$$L_{\text{total}} = \sum_{k=1}^{K} w_k \|G_k - O_k\|_2^2 + \lambda \left\| \phi_{\text{vgg}}(G_1) - \phi_{\text{vgg}}(O_1) \right\|_2^2, \qquad (S11)$$

where O_k and G_k denote the output of network and ground truth image at scale level k. Each scale is weighted with w_k, which are set as 1, 0.7 and 0.5 from large to small scale in our experiments. The second part of the loss function is the perceptual loss of the original image scale, calculated with the extracted features of the first 25 layers of VGG19 (*7*). The weight of perceptual loss λ is empirically set to 2×10^{-3}.

Dataset: To generate the training data, we calibrated sparse PSFs of the system from 0.03m to infinity. In practice, we fix the camera and place a point light source at different positions from 0.03m to 5m to capture the PSFs and calibrate the PSF at infinity with a collimator shown in Fig. S10. It could be found that there is no significant difference between the PSFs at 5 m and at infinity. With the PSF augmentation strategy, all PSFs in the extreme DoF could be covered in the training dataset. Considering the central symmetry property of optical system and error of calibration, the captured PSF set is augmented by randomly rotating, scaling and channel exchanging. The size of each PSF is set as 51×51. Specifically, we rotate and slightly resize the PSFs calibrated at different locations and depths randomly to generate the sparse PSF basis, and linearly combine the wavefront errors reconstructed from these sparse PSF basis using phase retrieval. The augmented dense PSFs could be computed by transforming the generated wavefront errors using Rayleigh-Sommerfeld diffraction in Eq. (3). We further add white Gaussian noise (with standard deviation from 0 to 5, bit-width of captured image is 8) to the training data to improve the noise robustness of the network. The ground truth sharp images are generated from COCO dataset (*8*) by randomly sampling 10,000 ground truth images. We convolve the ground truth images with the calibrated PSFs to generate the distorted images and then cut them randomly into patches with pixel size of 224×224 to train the neural network.

Training: The network is implemented upon the Pytorch platform. We train the network using the Adam optimizer (*9*) with $\beta_1 = 0.9$ and $\beta_2 = 0.999$. The total number of training epoch is set to be 50. The learning rate is initially set to 10^{-4} within the first 25 epochs and decayed to 10^{-5} in the last 25 epochs. The batch size is set to 4. The whole training procedure takes ≈ 10 hours on a commercial graphics processing unit (GPU, GeForce RTX 2080), with 2.2 M parameters.

Testing: Currently, the trained neural network requires 266 GFLOPS for inferencing a 512×512 image. Since currently the network is not optimized for computational efficiency, higher efficiency for real-time light-field imaging can be achieved by employing network acceleration methods, such as

quantification (*10-11*), network pruning (*12-13*) or other specially designed efficient architectures (*14-15*).

3. Disparity reconstruction method

Based on the distortion corrected light-field image, we further extract the disparity information via multi-view stereo matching (*16-17*), as shown in Fig. S7a. First, for two neighboring sub-images (Fig. S7b), a matching cost volume is calculated with the squared difference metric with respect to different possible disparities, which is further smoothed with a 3×3 average filter. Here the possible range of disparity is from 0 to the pixel number of sub-image. Based on this calculation approach, for each sub-image, 8 matching cost volumes are calculated with respect to its 8 different nearest neighbors (Fig. S7c). Then, the cost volume of sub-image is obtained by averaging these 8 cost volumes, and further regularized with a semi-global strategy (*18*) and refined by graph-cut (*19*). Finally, the disparity of each pixel is determined by the smallest cost value of the pixel.

4. Light-field rendering method

We implement the light-field rendering based on the framework described in Ref. (*20*). Center-view all-in-focus images are rendered in the following four steps: (1) extracting the center patch from each sub-image; (2) scaling extracted patches depending on the dominant disparity of each sub-image; (3) weighting the scaled patches with confidence maps; (4) tiling the weighted patches to a single image. The whole pipeline of the rendering method is illustrated in Fig. S8.

We first extract the center patch from each sub-image to eliminate the dark margin. Then we scale the patch based on the its dominant disparity *m* with the scaling factor,

$$k = \frac{d}{m}\,, \tag{S12}$$

where d is the patch size. With the scaling, the correspondences of different patches can be relocated to the same position.

To reduce the artifacts, we introduce a confidence weighting mask of each patch. The confidence of each pixel is inversely correlated with the difference between its disparity and the dominant disparity of the entire patch,

$$W_{\mathrm{conf}} = \max\left(e^{-C_1|m-\mathrm{disparity}|}, C_2\right), \tag{S13}$$

where W_{conf} is the confidence weighting mask, C_1 ,C_2 are both set to 0.1 in the experiments. After weighting the patches, we tile them together to get the final center-view image. To smooth the tiling artifact, a 2D Kaiser window is introduced to weight each patch. To generate refocused images at different depths, we introduce different Gaussian filters that correspond to different depths to blur each sub-images and generated the refocused images with the rendering method discussed above.

IV Experimental details

With the fabricated metalens array, we built a prototype bifocal light-field imaging system. The schematic and the prototype system are shown in Fig. S9. The system is composed of a commercial primary lens (focal length: 50 mm and aperture size: 6 mm), the designed metalens array (f_l: 0.90 mm, f_r: 1.25 mm, metalens size: 150 μm, 39×39 array of bifocal metalens) and a relay imaging system consisting of a 10× objective lens (Olympus RMX) and an imaging sensor (Grasshopper3 GS3-U3-41S4C). In addition, the experiment setups of capturing PSFs of object in the nearest position and infinity are shown in Fig. S10.

Figs. S11 shows the imaging scene for capturing Fig. 6. As shown, from nearest possible position at the front-end of the camera lens to far away houses, we are able to capture and reconstruct in-focus image of it. To further demonstrate the imaging capability of the proposed light-field imaging system. As shown in Fig. S12, we further capture images of the resolution chart at different depths, i.e., from 3 cm to infinity. To simulate the infinity object, here we take the image of the USAF-1951 resolution chart at infinity distance with a collimator (Figs. S12c-d). The aberration corrected light-field image and rendered center-view image of USAF1951 resolution chart at infinity are shown in Figs. S12e and f, respectively. Note that for object at infinity, spatial resolution is no longer meaningful and so we adopt the angular resolution to characterize the imaging performance (The captured images at the other distances are shown in Fig. 5 in the main text). The corresponding angular resolution of the metalens array-based camera with respect to depth is calculated and given in Fig. S13. As shown, from 3 cm to infinity, the resolution of imaging system matches well with the theoretical diffraction-limited ones calculated across the visible wavelength (rainbow region). These results further demonstrate the excellent imaging capability which has never been achieved before.

Fig. S14 shows the imaging results of the scene covering a depth from 3 cm to 1700 m without aberration correction for different polarization channels. For the LCP channel, the camera can obtain in-focus image for the near objects, such as the 'NJU' string and ruler, but out-of-focus image for far objects (Fig. S14a). As expected, when the polarization state of light is switched from LCP to RCP, the system can obtain in-focus image for the far objects like color plate, university logo and buildings, but instead acquires the out-of-focus image for the near objects, i.e. the string and ruler (Fig. S14b). For operation in natural light, the camera can simultaneously obtain in-focused image for both near and far objects (Fig. S14c). The image in natural light is more blurred than that in a single polarization state due to cross-talk between the two polarization imaging channels. However, since the crosstalk could be regarded as low-frequency noise added to the clear image, it can be eliminated using our aberration correction algorithm. Due to the limited field-of-view of the relay lens, here we capture 4×4 views and tile them together to obtain the large field-of-view of image.

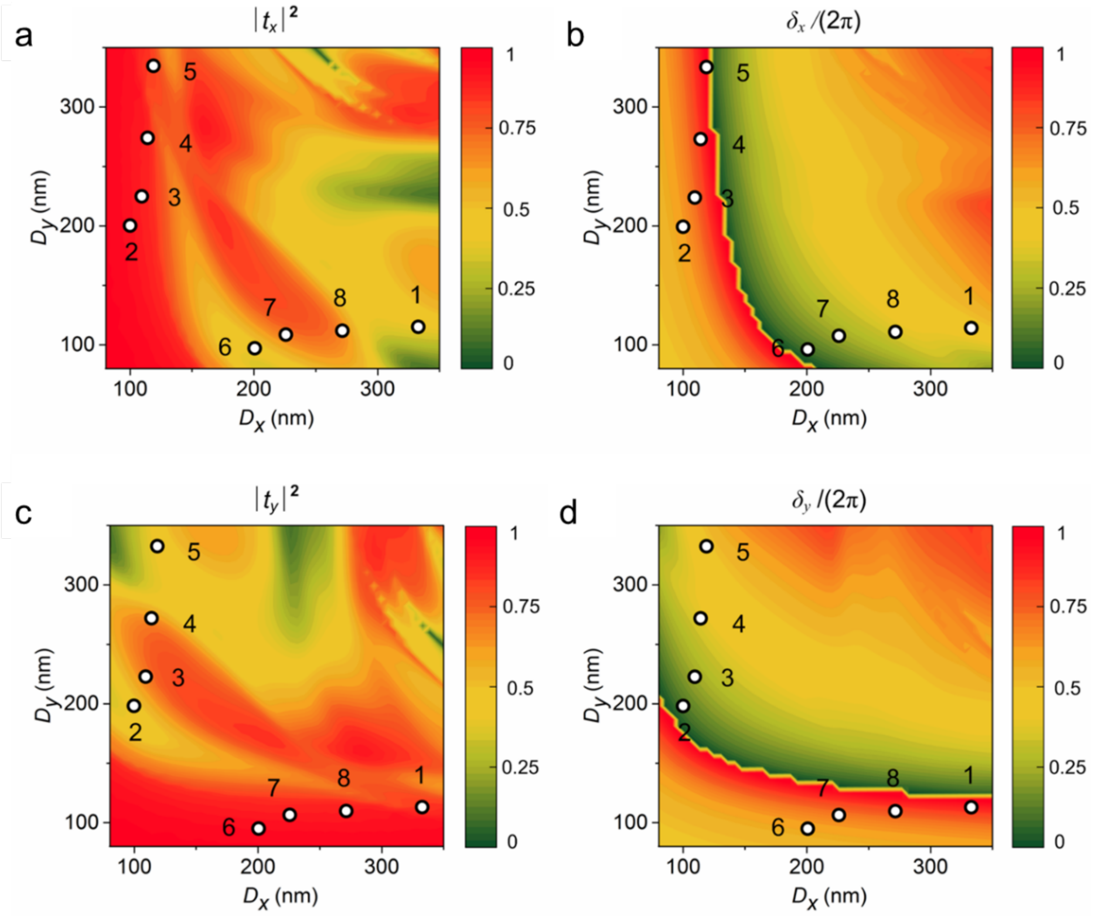

Figure S1 | Calculated intensity (a.$|t_x|^2$; c. $|t_y|^2$) and phase shifts (b. δ_x; d. δ_y) of transmission coefficients as a function of axis length (D_x, D_y) of the rectangular nanopillar at the wavelength of 530 nm. The white dots indicate four fundamental nanostructures #1 to #4: #1(335 nm, 120 nm), #2(100 nm, 200 nm), #3(110 nm, 225 nm), #4(115 nm, 270 nm). By rotating the nanopillars by an angle of 90°, the other four mirror structures #5 to #8 can be obtained.

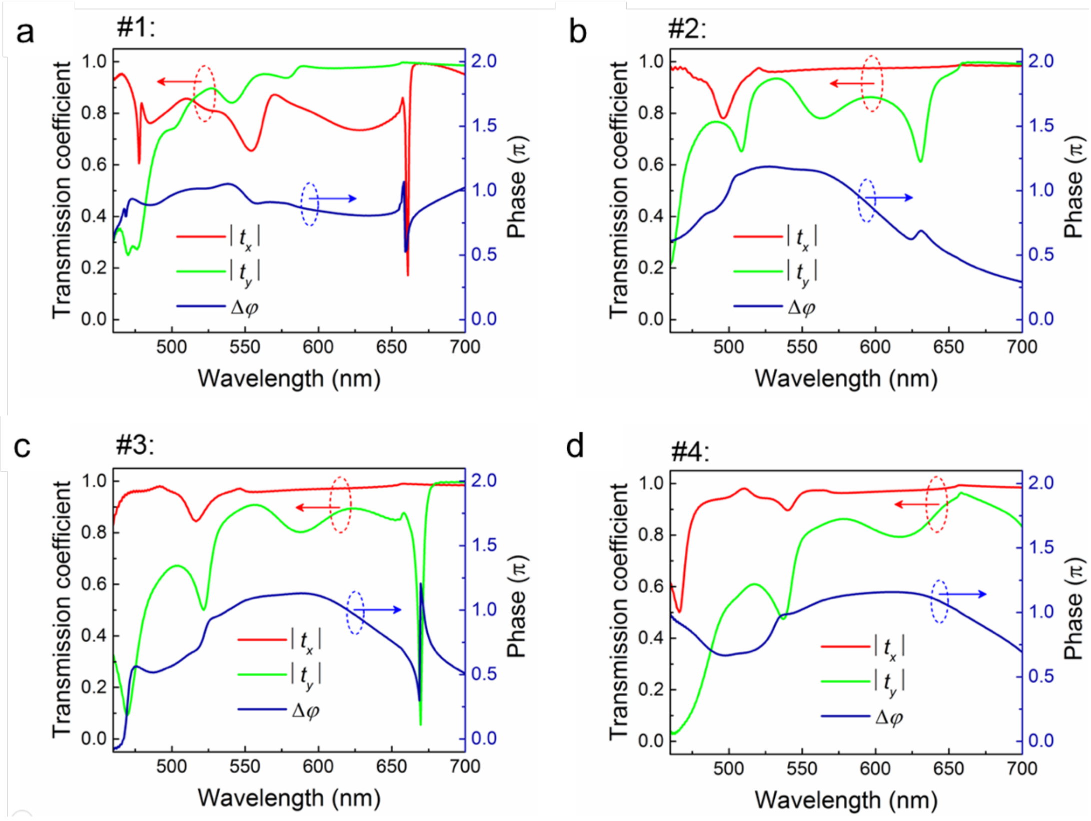

Figure S2 | Simulated transmission coefficients (red $|t_x|$; green $|t_y|$) for *x*- and *y*-polarized light and their phase difference (blue, $\Delta\varphi$) over the entire visible spectral range (460 nm - 700 nm).

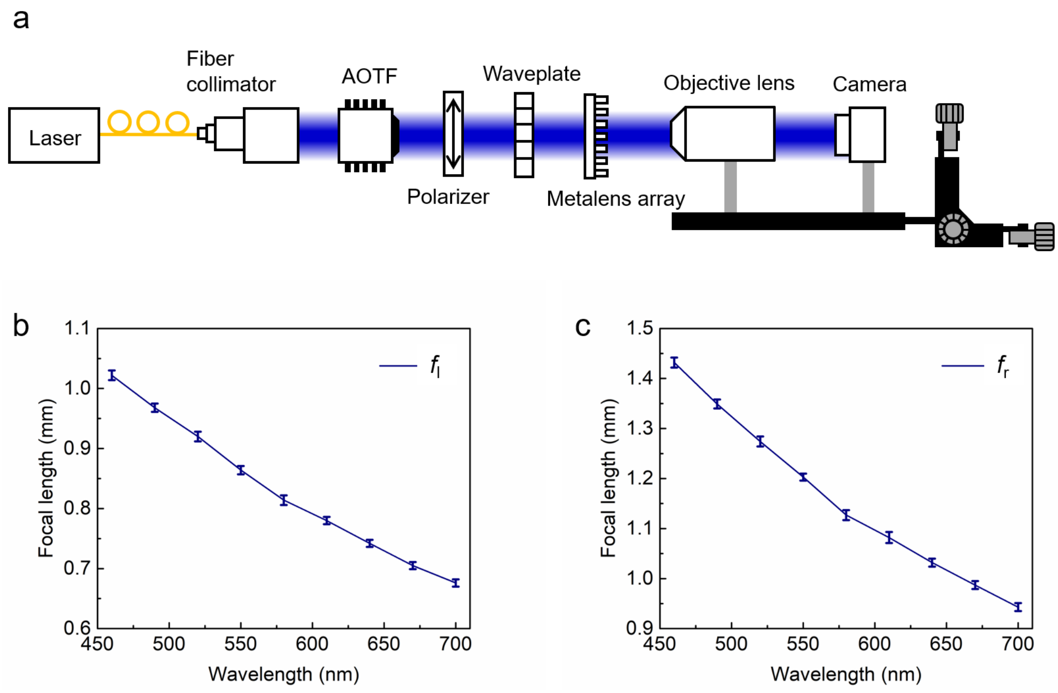

Figure S3 | Metalens array characterization. (a) Experimental setup for focal spot measurements. A collimated beam passes through a polarizer and quarter-wave plate to generate circularly polarized light. (b, c) Measured focal lengths. The uncertainties are standard deviation for repeated experimental measurements (four in total).

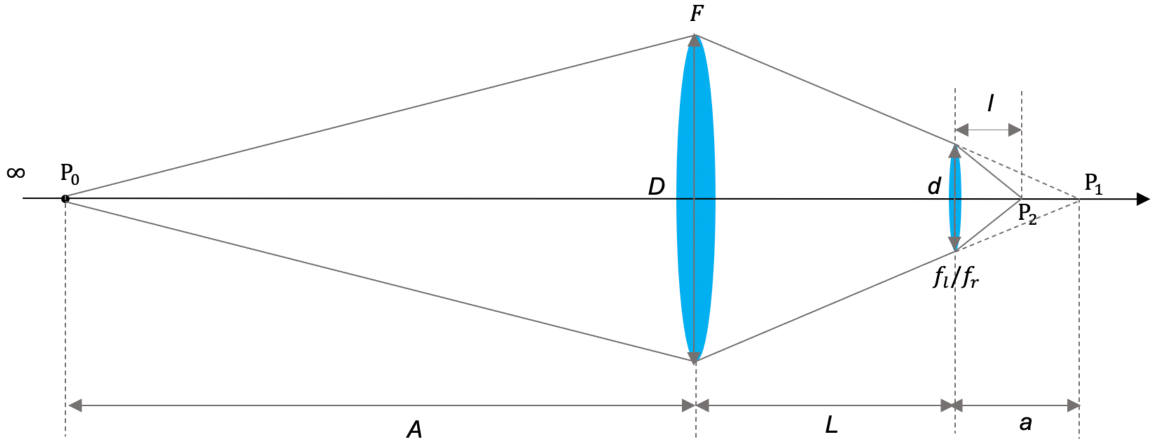

Figure S4 | Simplified geometric scheme of the imaging system. The object point P_0 is focused by the primary lens to the image point P_1, and the image point P_1 is further focused by the metalens to the image point P_2.

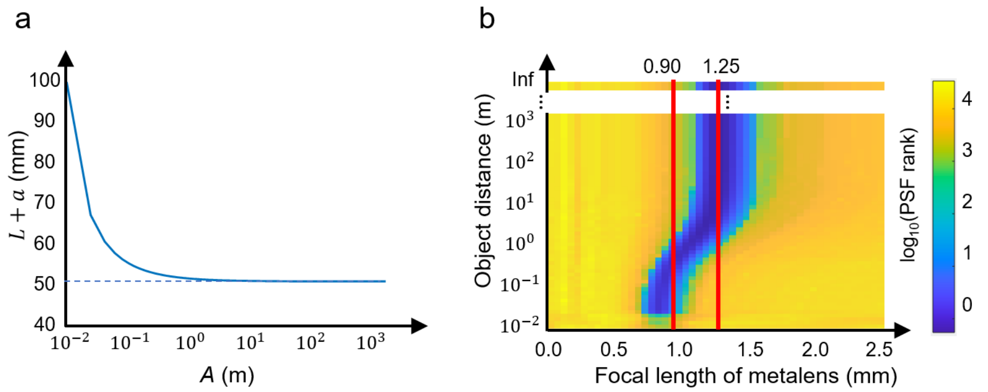

Figure S5 | Parameter chosen analysis of $L + a$ and f_l. (a) The relationship between $L + a$ and the object distance A. (b) The PSF rank distribution with different focal length of meta-lens at different object distances. Note that here the object distance is shown in log scale.

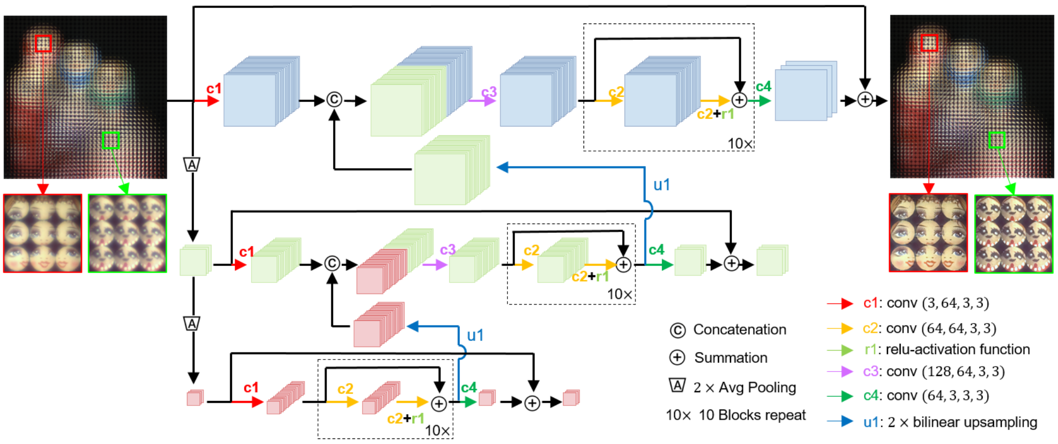

Figure S6 | Architecture of the distortion removal convolutional neural network. The network is composed of 3 branches in different scales. Each branch of network is composed of residual blocks, convolutional blocks, skip connections. The feature information of different scales are elegantly fused together with convolutions and concatenations among different network branches.

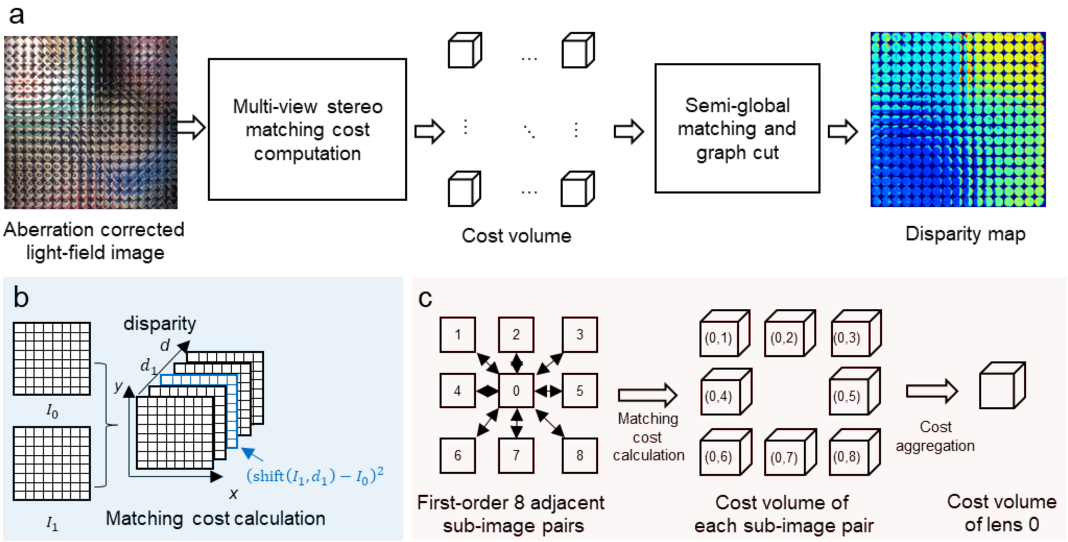

Figure S7 | The schematic of the disparity estimation method. (a) The overall disparity extraction pipeline. (b) Illustration of the cost volume calculation method among two sub-images and (c) the cost volume calculation of multi-view stereo matching algorithm.

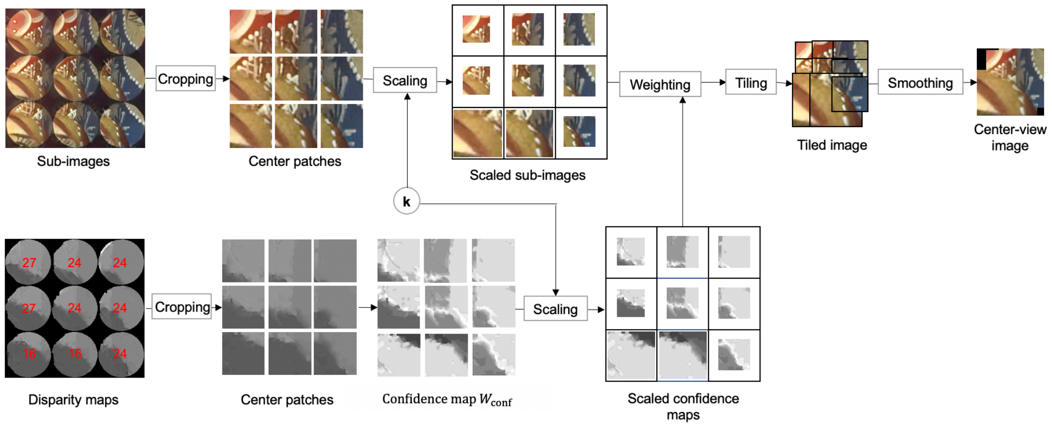

Figure S8 | Pipeline illustration of the rendering method. Note that here we take a 3 × 3 sub-images as example. Firstly, the center patch of each sub-image is extracted through cropping. Then the extracted patches are rescaled depending on the dominant disparity of each sub-image. Thirdly, the rescaled patches are weighted with the confidence maps calculated with the disparity map. Finally, the weighted patches are tiled together into a rendered center-view image.

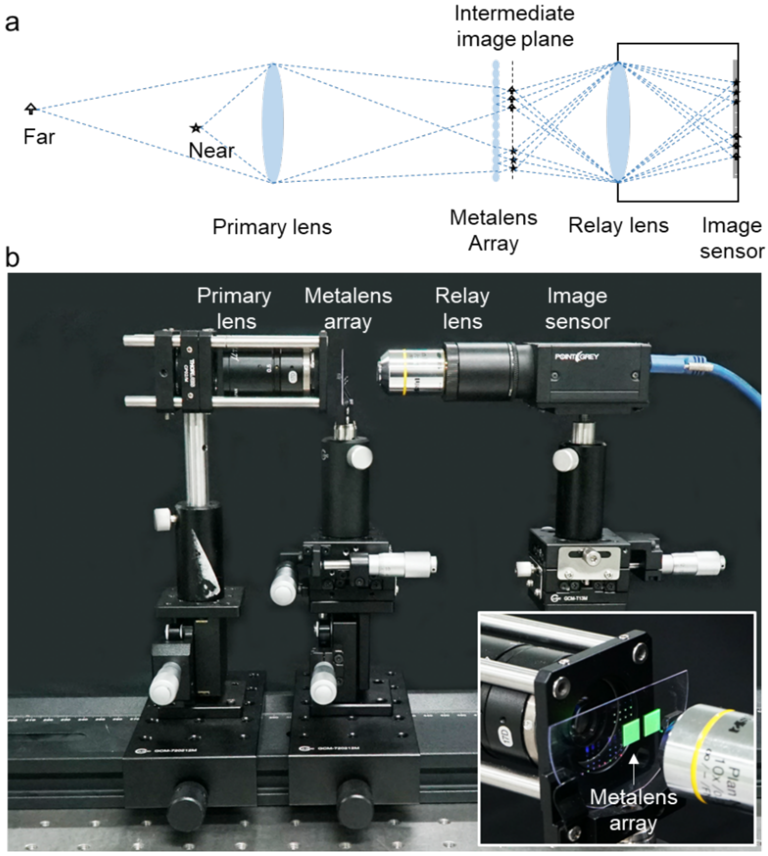

Figure S9 | Imaging system. (a) Schematic of the prototype bifocal light-field imaging system with relay system. Note that for clarity, for each object ('star' or 'tree'), only the chiral light that focused at the image sensor plane is plotted out. (b) Image of the proposed prototype light-field imaging system and the inset shows the proposed metalens array.

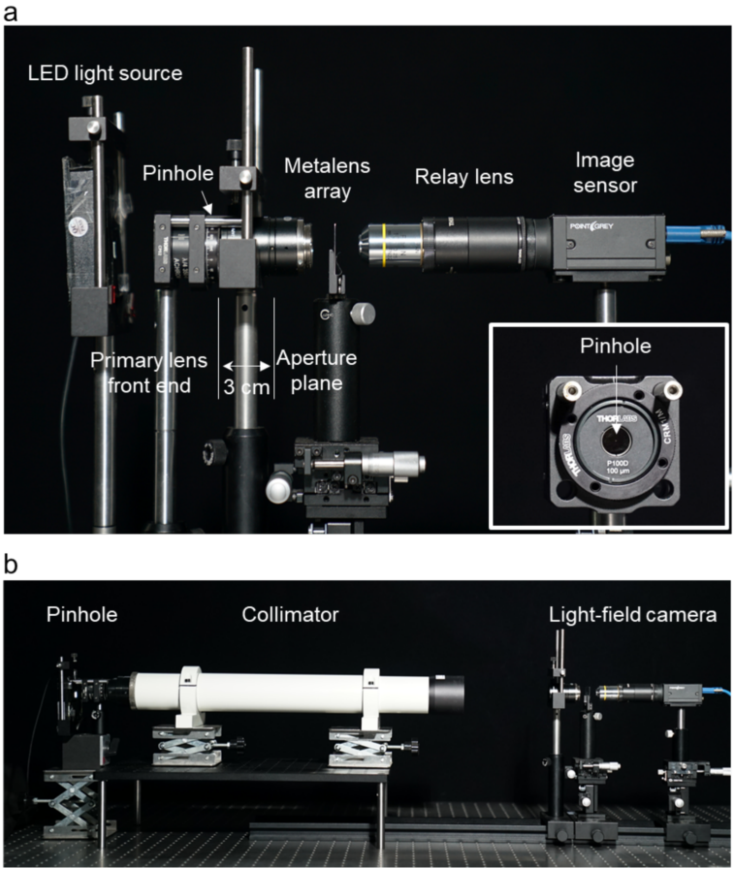

Figure S10 | (a) The imaging scheme of capturing the nearest possible PSF at 3 cm distance. Note that when the pinhole is right at the front end of the main lens, the distance between the pinhole and the aperture of the main lens is 3 cm. (b) The imaging scheme of capturing the point spread function at infinity with the collimator.

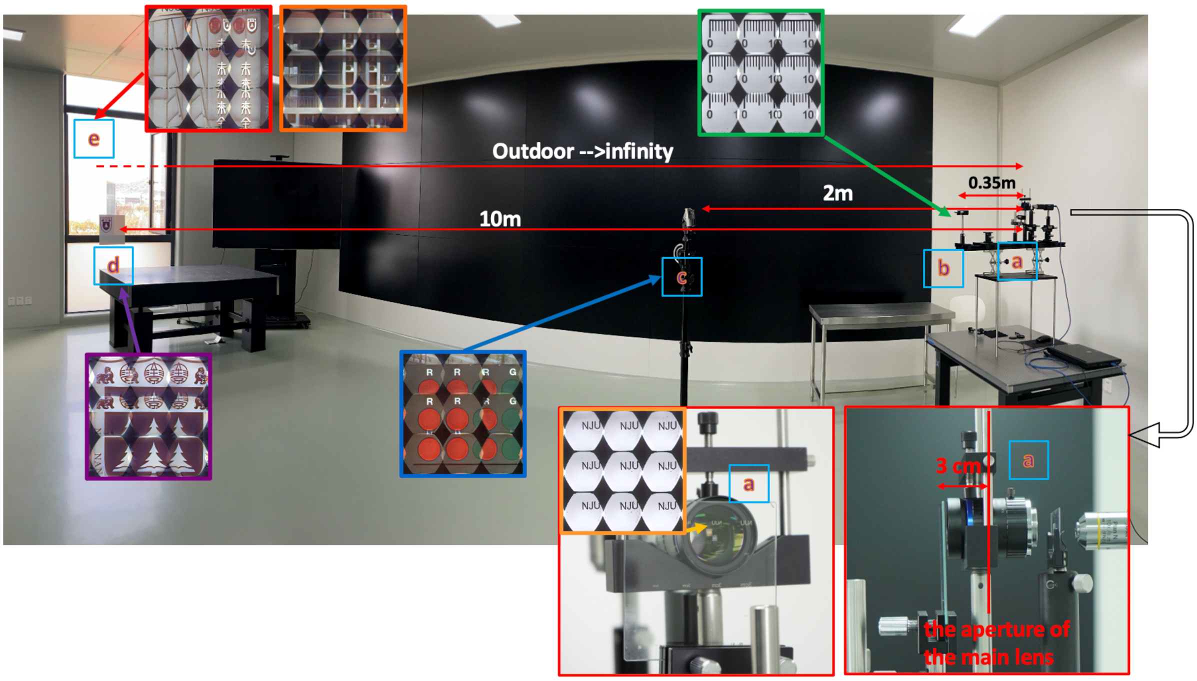

Figure S11 | The overview of the imaging scene of Fig. 6 (in the main text). The scene is composed of objects at different distances from the aperture of the primary lens, *i.e.*, (a) 3 cm 'NJU' letters, (b) 0.35 m ruler, (c) 2 m color plate, (d) 10 m Nanjing University Logo, and (e) the outdoor scene.

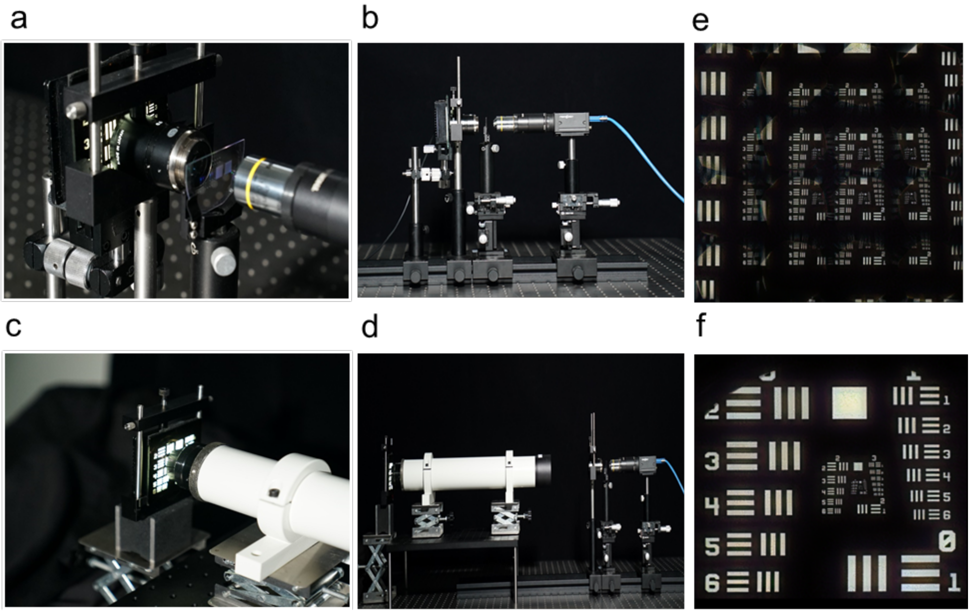

Figure S12. (a-b) The imaging system that focused at 3cm, which is right at the front-end of the primary lens. (c-d) The imaging system that focused at infinity through a collimator. (e-f) The aberration corrected light-field and center-view rendered USAF1951 image at infinity.

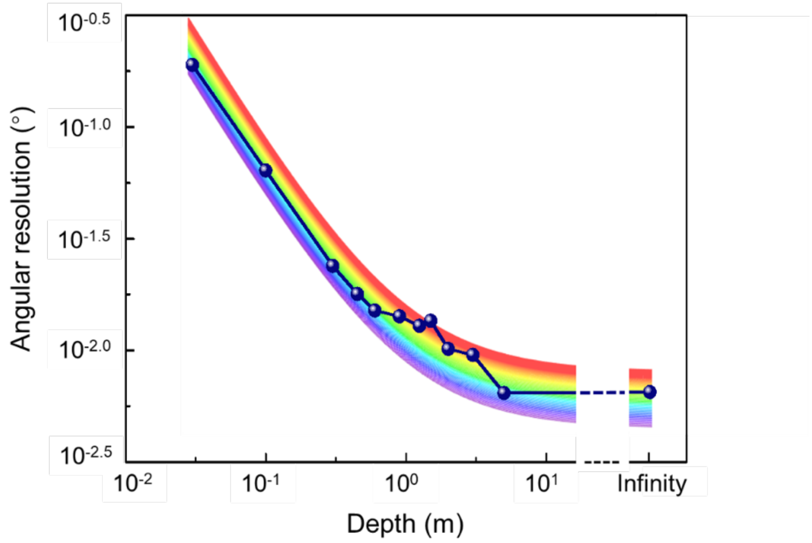

Figure S13 | Experimentally measured (blue sphere) and theoretical (rainbow region) angular resolution of the light-field camera. Blue to red colors for the theoretical results correspond to the visible wavelength.

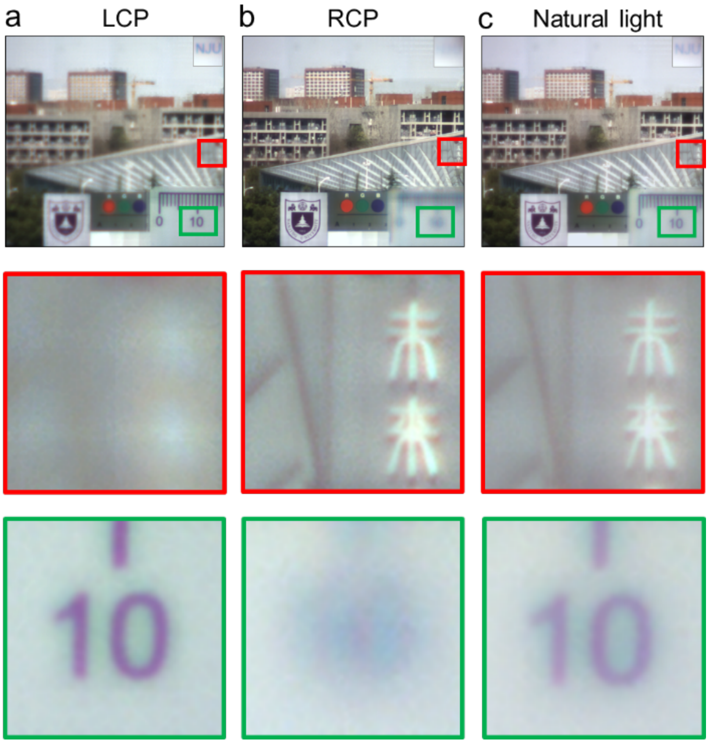

Figure S14 | The rendered center-of-view images for LCP (a), RCP (b), and natural light (c) without applying aberration correction algorithm. The images in red and green wireframes show the magnified distant scene and close scene, respectively. The white Chinese characters ‘future’ on the rooftop are about 360 m away and the ruler is placed at the depth of 0.35 m. The smallest scale of the ruler is 1 mm. The reconstructed NJU characters have been reasonably shifted and scaled for easy viewing.

References

1. J. Gal, G. Horvath, E. N. K. Clarkson, O. Haiman, Image formation by bifocal lenses in a trilobite eye? *Vision Res.* **40**, 843–853 (2000).
2. J. Li, F. Fang, K. Mei, G. Zhang, Multi-scale Residual Network for Image Super-Resolution, *Proceedings of the European Conference on Computer Vision*, 517-532 (2018).
3. S. Gu, Y. Li, L. V. Gool, R. Timofte, Self-guided network for fast image denoising, *Proceedings of the IEEE International Conference on Computer Vision*, 2511-2520 (2019).
4. S. Nah, T. Hyun Kim, K. Mu Lee, Deep multi-scale convolutional neural network for dynamic scene deblurring, *Proceedings of the IEEE Computer Vision and Pattern Recognition*, 3883-3891 (2017).
5. K. He, X. Zhang, S. Ren, J. Sun, Deep residual learning for image recognition, *Proceedings of the IEEE Computer Vision and Pattern Recognition*, 770-778 (2016).
6. L. A. Gatys, A. S. Ecker, M. Bethge, Image style transfer using convolutional neural networks, *Proceedings of the IEEE Computer Vision and Pattern Recognition*, 2414-2423 (2016).
7. K. Simonyan and A. Zisserman, Very deep convolutional networks for large-scale image recognition, *Proceedings of International Conference on Learning Representations*, 1-14 (2015).
8. https://cocodataset.org/
9. D. P. Kingma, J. Ba, Adam: A method for stochastic optimization, *Proceedings of International Conference on Learning Representations*, 1-15 (2015).
10. S. Jung, C. Son, S. Lee, J. Son, J. J. Han, Y. Kwak, S. J. Hwang, C. Choi, Learning to quantize deep networks by optimizing quantization intervals with task loss. *Proceedings of the IEEE Conference on Computer Vision and Pattern Recognition*, 4350-4359, (2019).
11. J. Liu, B. Zhuang, Z. Zhuang, Y. Guo, J. Huang, J. Zhu, M. Tan, Discrimination-aware network pruning for deep model compression. *IEEE Trans. Pattern Anal. Mach. Intell.* (2021).
12. F. Tung, G. Mori. Deep neural network compression by in-parallel pruning-quantization. *IEEE Trans. Pattern Anal. Mach. Intell.* **42**, 568-579 (2018).
13. S. Chen, Q. Zhao, Shallowing deep networks: layer-wise pruning based on feature representations. *IEEE Trans. Pattern Anal. Mach. Intell.* **41**, 3048-3056 (2018).

14. A. G. Howard, M. Zhu, B. Chen, D. Kalenichenko, W. Wang, T. Weyand, M. Andreetto, H. Adam, Mobilenets: Efficient convolutional neural networks for mobile vision applications. *arXiv preprint arXiv:1704.04861* (2017).
15. X. Zhang, X. Zhou, M. Lin, J. Sun, Shufflenet: An extremely efficient convolutional neural network for mobile devices. *Proceedings of the IEEE conference on computer vision and pattern recognition* 6848-6856, (2018).
16. O. Fleischmann, R. Koch, Lens-based depth estimation for multi-focus plenoptic cameras, *Proceedings of the German Conference on Pattern Recognition*, 410-420 (2014).
17. L. Palmieri, R. Koch, R. O. H. Veld, The Plenoptic 2.0 Toolbox: Benchmarking of depth estimation methods for MLA-Based focused plenoptic cameras, *Proceedings of the IEEE International Conference on Image Processing*, 649-653 (2018).
18. H. Hirschmuller, Stereo processing by semi-global matching and mutual information, *IEEE Trans. Pattern Anal. Mach. Intell.* **30**, 328-341 (2007).
19. Y. Boykov, V. Kolmogorov, An experimental comparison of min-cut/max-flow algorithms for energy minimization in vision. *IEEE Trans. Pattern Anal. Mach. Intell.* **26**, 1124-1137 (2004).
20. T. Georgiev, A. Lumsdaine, Focused plenoptic camera and rendering. *J. Electronic Imaging* **19**, 021106 (2010).
